# Gaseous cues regenerate the periderm

**DOI:** 10.1016/j.xplc.2025.101576

**Published:** 2025-10-27

**Authors:** Hassan Iqbal, Chen Yaning, Muhammad Waqas, Tom Beeckman, Ülo Niinemets, Christoph-Martin Geilfus

**Affiliations:** 1 State Key Laboratory of Desert and Oasis Ecology, Xinjiang Institute of Ecology and Geography, Chinese Academy of Sciences, Urumqi 830011, China; 2 Xinjiang Laboratory of Lake Environment and Resources in Arid Zone, College of Geographic Science and Tourism, Xinjiang Normal University, Urumqi, China; 3 Department for Plant Nutrition and Soil Science, Hochschule Geisenheim University, 65366 Geisenheim, Germany; 4 Chair of Plant and Crop Science, Estonian University of Life Sciences, Kreutzwaldi 1, 51006 Tartu, Estonia; 5 Department of Plant Biotechnology and Bioinformatics, Ghent University, Technologiepark 71, 9052 Ghent, Belgium; 6 VIB-UGent Center for Plant Systems Biology, Technologiepark 71, 9052 Ghent, Belgium

## How do plants sense injury and regenerate the periderm?

All living organisms rely on protective tissues, just as human skin shields us from environmental stresses and heals after injury. Many plants, particularly woody species, possess a multilayered outer tissue known as the periderm, which originates from the secondary lateral meristem phellogen and replaces the epidermis. This protective barrier defends against pathogens, dehydration, and mechanical damage, and its regeneration from dedifferentiated parenchyma cells following injury is vital for plant survival (Serra et al., 2022). Researchers have long recognized that certain hormones, particularly ethylene and jasmonates, activate wound-healing responses in plants (Zhang et al., 2019; Zhao et al., 2021; Serra et al., 2022). However, the mechanism by which plants initially detect breaches in their protective barrier remains unclear. Gaseous molecules such as oxygen and ethylene are known to regulate a wide range of developmental and stress-related processes, and they diffuse passively through plant tissues. Could alterations in their diffusion dynamics act as early injury signals that initiate periderm regeneration? This concept has not been explored and has remained an open question for decades, although recent advances have begun to shed light on this mechanism.

## Ethylene and oxygen as binary switches in wound healing

**Figure 1:**
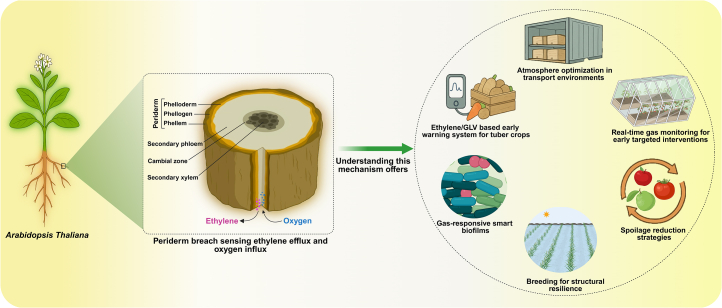
Periderm breach sensing in *Arabidopsis* roots and its potential applications *A. thaliana* serves as a model for studying periderm integrity, where tissue breaches trigger ethylene (C_2_H_4_) efflux and oxygen (O_2_) influx. Understanding this gas-exchange mechanism provides translational opportunities, including ethylene/GLV-based early warning systems for tuber crops, atmosphere optimization during transport, real-time gas monitoring for precise interventions, spoilage reduction strategies, development of gas-responsive smart biofilms, and breeding approaches aimed at enhancing structural resilience in crops.

A recent groundbreaking study in *Arabidopsis thaliana* (*A. thaliana*) by Iida et al. (2025) advanced our understanding of plant wound biology by revealing that barrier integrity can be monitored through the passive diffusion of ethylene (efflux) and oxygen (influx), which act as binary switches to trigger regeneration (Figure 1). While earlier studies suggested that ethylene production increases after injury, this work reveals that reduced ethylene signaling is, in fact, crucial for initiating wound healing. The researchers induced damage by cutting *A. thaliana* root tissues and monitored the responses using reporter assays and mutant analyses. Within 24 h of injury, cork (phellem) development markers such as *PER15* were activated near the wound site. After 48 h, phellogen formation began, accompanied by periclinal cell divisions. Expression of *WOX4*, a key regulator of procambial development, was clearly upregulated by the third day, and after 96 h, a fully developed, lignified, and suberized barrier resembling mature phellem had restored the tissue’s protective function (Iida et al., 2025).

Iida et al. (2025) further investigated whether stress hormones influence periderm regeneration. Among several candidates, the ethylene precursor ACC (1-aminocyclopropane-1-carboxylate) suppressed *PER15* expression after injury, and ethylene treatment produced similar inhibitory effects. In ethylene-insensitive mutants, ACC failed to block *PER15* activation, confirming that ethylene signaling suppresses periderm-related gene induction (Iida et al., 2025). Histological staining showed that ACC and ethylene treatments disrupted suberin deposition, resulting in discontinuous and leaky periderm layers (Iida et al., 2025). Gas chromatography confirmed a sharp increase in ethylene release from wounded roots, whereas sealing wounds with lanolin/Vaseline maintained high ethylene signaling and inhibited regeneration (Iida et al., 2025). These results demonstrate that ethylene must diffuse away from the wound site to reduce local signaling, an essential reduction for activating the periderm repair program (Iida et al., 2025).

However, ethylene alone does not fully explain the process, as ACC treatment did not completely suppress periderm formation at the wound site, suggesting the involvement of additional gaseous compounds. One likely candidate is oxygen, which may enter through the wound and facilitate periderm regeneration. Oxygen is necessary for the suberization of periderm cell walls, a process mediated by peroxidases such as *PER15* and reactive oxygen species (Serra and Geldner, 2022). Indeed, the downregulation of hypoxia-related genes *PCO1* and *PCO2* during wound healing confirms the enhanced oxygen entry through the wound (Iida et al., 2025). To verify this, the authors used oxygen microsensors to measure O_2_ dynamics in both intact and wounded roots. They also combined ACC treatment with hypoxia-related mutants to test the additive effects of ethylene and oxygen signaling. The results revealed that ethylene diffusion and oxygen entry at wound sites coordinately trigger periderm regeneration through reduced ethylene and hypoxia signaling. As new periderm forms and the wound closes, gas diffusion slows, restoring ethylene signaling and hypoxic conditions, which in turn terminate the regeneration process (Iida et al., 2025). This binary role of gas flux, as both initiator and inhibitor, represents a self-regulating feedback system in *Arabidopsis* root tissues.

Beyond periderm damage, Iida and colleagues (Iida et al., 2025) also investigated *Arabidopsis* stems, which lack periderm and rely on the epidermis as the primary environmental barrier. Wounded stems formed phellem-like suberized layers and exhibited increased ethylene emission, similar to roots. However, unlike in roots, ethylene signaling mutants showed normal barrier reformation, and hypoxia-dependent signaling played no detectable role. Sealing the wound still prevented regeneration, indicating that gas diffusion remains important but involves distinct molecular players compared with roots (Iida et al., 2025). This difference may reflect the greater oxygen availability in the aerial tissues of *Arabidopsis*. In general, aerial tissues of herbaceous species possess stomata similar to those in leaves. *Arabidopsis* stems are well aerated, which may explain why hypoxia-related signaling was not detected there. By contrast, in many woody species, stems develop a thick periderm that restricts gas diffusion and reduces oxygen levels near wounds. To test how gas diffusion influences regeneration, it will be informative to compare systems across a gradient of oxygen availability and barrier thickness, including *Arabidopsis* stems without a true periderm, woody stems with a thick periderm, and roots of varying periderm thickness. The tissue-specific variation observed in *Arabidopsis* raises two key questions. First, which volatile compounds act together with ethylene and oxygen to re-establish the barrier in stems? Green leaf volatiles (GLVs) are strong candidates, as they are produced via the oxylipin pathway following activation of lipoxygenases (Ameye et al., 2018) and diffuse rapidly. C5 and C6 denote GLVs with five or six carbon atoms, for example, pentenol and pentenyl acetate for C5, and hexenal, hexenol, and hexenyl acetate for C6. GLVs are rapidly released after mechanical injury and are primarily known as signaling molecules that activate a wide range of defense responses (Engelberth, 2024). However, their role in wound healing remains surprisingly underexplored. Nevertheless, GLV release is associated with the production of traumatin—a wound hormone that promotes secondary meristem formation (Engelberth, 2024). Lipoxygenases, which catalyze the initial steps of GLV and traumatin biosynthesis, depend on oxygen availability. Therefore, investigating how increased oxygen levels resulting from wounding contribute to barrier reformation and periderm regeneration represents a promising avenue for future research.

Although monocot crops such as cereals lack a true periderm, they possess ethylene signaling pathways distinct from those of dicots (Zhao et al., 2021; Kong et al., 2024). This raises the question of whether cereals rely on alternative gas-sensing strategies to repair tissue after injury. The idea that limited gas flow could act as a regulatory signal for healing is intriguing, yet whether this self-regulating gas feedback mechanism operates in monocots remains untested. Further studies are needed to determine whether such responses contribute to stress adaptation in cereals. In rice, compacted soils impose mechanical stress on the outer root layers while simultaneously limiting gas exchange. Waterlogged conditions further increase ethylene levels, which regulate key processes such as aerenchyma formation and internode elongation (Zhao et al., 2021; Kong et al., 2024). Given that ethylene signaling mutants in rice show profiles distinct from those in *Arabidopsis*, these genotypes serve as valuable models to test whether gas dynamics play similar roles in monocots. Ultimately, such insights may inform breeding strategies aimed at enhancing resilience to soil compaction and flooding.

## From wound to yield: Harnessing gas sensing for sustainable food security

The discovery that gas diffusion regulates wound healing opens new opportunities for developing targeted strategies to enhance tissue repair in economically important crops (Figure 1). For instance, the shelf life of root vegetables such as potato and carrot, both of which form a periderm, depends on the integrity of their protective tissues. These crops could benefit from controlled atmosphere approaches, such as modified atmosphere treatments or soil-applied ethylene inhibitors, designed to fine-tune gas-sensing and accelerate barrier regeneration. Such interventions may be especially effective in controlled environments such as greenhouses or tunnels, where ethylene and oxygen levels can be precisely managed. During postharvest handling, damage to protective barriers can cause moisture loss, microbial decay, reduced nutritional quality, and ultimately spoilage. Similarly, fruit crops such as apple and pear, which form wound periderm in response to skin damage, could be engineered or treated to reduce spoilage-related losses that currently amount to billions of dollars annually. To put this in perspective, postharvest losses account for nearly 40% of global food waste, emphasizing the importance of strategies that maintain tissue integrity.

The urgency for such innovations is intensified by climate change, which increases mechanical damage from storms, hail, and drought. Global warming also drives insect outbreaks that further damage crops and reduce the yields of cereals, legumes, and leafy vegetables. Recent advances reveal that cellular injury activates mechano-chemical pathways that regulate cell wall dynamics and patterned cell divisions during plant healing, with ethylene pathways playing a crucial role in maintaining proper division patterns (Di Fino et al., 2025). Combined with the gas-sensing mechanism uncovered by Iida et al. (2025), this mechanistic insight offers new opportunities to engineer crops with enhanced regenerative capacity by identifying key genes, such as *PER15*, *PER49*, *WOX4*, and members of the RAX family (*RAX1/MYB37*, *RAX2/MYB38*, and *RAX3/MYB84*; Wunderling et al., 2018; Iida et al., 2025). These genes could serve as molecular markers for selecting crop varieties with superior wound responses, vital for maintaining root integrity under drought stress and protecting shoots from mechanical damage (Figure 1).

These findings could lead to practical improvements in crop storage and transportation. Controlled atmosphere systems in warehouses or transport containers could be adjusted to not only maintain dormancy or ripening but also promote natural healing processes after harvest through short curing before storage, potentially extending the shelf life. Balancing oxygen and ethylene levels may enhance the suberization and repair of damaged surfaces, thereby reducing spoilage and improving postharvest longevity. At a larger scale, understanding gas diffusion within plant tissues could inform more targeted strategies in precision agriculture. Real-time monitoring of GLV and ethylene emissions could act as an early warning system for barrier damage, enabling timely and localized interventions. In this context, the development of gas-responsive smart biomaterials, such as agricultural films or sensors capable of detecting ethylene and/or GLV gradients, holds potential for adaptive field-level crop management (Figure 1). Such technologies have already been prototyped using multiplexed nanosensors that decode stress signals in real time (Ang et al., 2024). Another intriguing question is whether exposure of undamaged plant tissues to signaling molecules such as GLVs could induce enhanced periderm development, among other protective responses (Matsui and Engelberth, 2022). If so, this would open exciting possibilities for noninvasive targeted modulation of periderm thickness, a potentially cost-effective means of improving the shelf life of key root vegetables.

Taken together, the implications of this wound-healing discovery extend well beyond basic plant biology, providing a novel conceptual framework for improving crop resilience, postharvest preservation, and ultimately global food security. However, further efforts are needed to determine whether this gas-regulated repair mechanism is broadly conserved across diverse and economically important crop species.

## Funding

We sincerely acknowledge the financial support from the National Natural Science Foundation of China (grant no. 52161145102), the 10.13039/501100018537Ministry of Science and Technology of China through the Foreign Young Talent Program (project no. QN2022045008), and the International Partnership Program of the 10.13039/100030917Chinese Academy of Sciences (grant no. 131965KYSB20210045). Their contributions are gratefully appreciated.

## Acknowledgments

No conflict of interest declared.
